# An Experimental Investigation of Human Presence and Mobile Technologies on College Students’ Sun Protection Intentions: Between-Subjects Study

**DOI:** 10.2196/13720

**Published:** 2019-08-26

**Authors:** Zhaomeng Niu, David C Jeong, Elliot J Coups, Jerod L Stapleton

**Affiliations:** 1 Rutgers Cancer Institute of New Jersey New Brunswick, NJ United States; 2 University of Southern California Los Angeles, CA United States; 3 Rutgers Robert Wood Johnson Medical School New Brunswick, NJ United States

**Keywords:** skin cancer, mHealth, education, sunscreens

## Abstract

**Background:**

Health promotion and education programs are increasingly being adapted and developed for delivery through digital technologies. With this shift toward digital health approaches, it is important to identify design strategies in health education and promotion programs that enhance participant engagement and promote behavior change.

**Objective:**

This study aimed to examine the impact of an experiment testing various mobile health (mHealth) skin cancer prevention messages on sun protection intentions and message perceptions among American college students.

**Methods:**

A sample of 134 college students aged 18 years or older participated in a 2×2×2 between-subjects experimental study, designed to examine the individual and combinatory effects of multiple dimensions (human presence, screen size, and interactivity) of digital technologies. The primary study outcome was intention to use sun protection; secondary outcomes included attitudes toward the information, two dimensions of trust, and information processing.

**Results:**

Generally, intention to use sun protection was positively associated with the presence of human characters in the health educational messages (*P*<.001), delivering educational health messages on a large screen (ie, iPad; *P*<.001), and higher interactivity (*P*<.001). Only human presence produced more favorable attitudes (*P*=.02). Affective trust was positively associated with human presence (*P*=.006) and large screen size (*P*<.001), whereas cognitive trust was positively associated with human presence (*P*<.001) and small screen size (*P*=.007). Moreover, large screen size led to more heuristic processing (*P*=.03), whereas small screen size led to more systematic processing (*P*=.04).

**Conclusions:**

This experimental study demonstrates that the impact of mHealth skin cancer prevention messages differs based on platform and delivery design features. Effects on behavioral intentions, attitudes, and trust were found for conditions with human presence, highlighting the importance of including this feature in mHealth programs. Results from this experimental study can be used to optimize the design of mHealth educational interventions that promote sun protection.

## Introduction

Skin cancer is the most common cancer in the United States [1], and ultraviolet (UV) exposure has been found to be significantly positively associated with skin cancer. Indoor tanning and outdoor tanning caused by UV exposure are highly prevalent among college students [2]. Previous research has demonstrated that the knowledge level of skin cancer and sun prevention behaviors is low [3], which may lead to harmful outcomes such as sunburns and sun damage. There is a widely recognized need for health interventions to reduce skin cancer risk behaviors in young adult populations [4]. More than 85% of college students regularly use a smart device [5], and most of them have positive attitudes and high acceptance of mobile learning [6,7]. Thus, mobile digital technologies represent a potentially impactful approach to educate college students about the harms of tanning and importance of sun protection. However, there is a dearth of research about how to best design mobile health (mHealth) educational interventions using both technological features along with social elements in messages. This study used an experimental design to examine the impact of manipulating design aspects of a website promoting sun protection on recipients’ message engagement and intentions.

College students today use digital technologies for both social (networking) and academic (learning) purposes [8], warranting the application of social elements of technologies when designing educational messages. Social cognitive theory posits that individuals could learn knowledge and behaviors by following a role model within a particular social context [9,10]. Following a model may motivate individuals to perform behaviors they are less familiar with or those that were more recently learned [11]. For instance, the social presence of humans in food advertising, as opposed to objects, has been found to have a positive influence on eating behaviors and food choices through body image and norms [12,13]. Although the importance of applying social elements in health intervention is widely recognized [14,15], few studies have been designed to specifically examine the impact of human presence in health interventions. This study aimed to explore the influences of presence of human characters in health educational messages on behavioral intentions and information processing.

The impact of messages on intentions and processing also depends on the way that information is presented on mobile devices [16]. One critical form–based factor of mobile devices that has impacted viewers’ media experiences is screen size [17,18]. Different screen sizes of mobile devices may impact information processing and different dimensions of trust [17-20]. Heuristic processing described the type of message process that is based on judgmental cues and requires less cognitive effort to process information and make decisions. Systematic processing represents the use of more cognitive effort, such as knowledge and attention, when processing messages [21]. Understanding these mechanisms on different mobile devices can help understand how information process affects the outcomes of persuasive communications through the mobile platform. Affective trust is an emotion-driven trust based on personal bonds or feelings and is often measuring trust from an emotional perspective with adjectives that describe feelings such as *likable* and *warm*. Cognitive trust is a logic-driven trust and is related to judgments of reliability of information [22]. In a previous study, large-screen smartphones lead to higher amount of heuristic processing and affective trust, whereas small-screen smartphones resulted in more comprehensive, systematic information processing and cognitive trust in advertising [20].

Another unique feature of digital technologies is the interactivity function of computers or mobile devices. Modality interactivity refers to the incorporation of interactive tools that afford users greater activity onto medium interfaces [23] and has been demonstrated as effective for users [24], particularly in the domain of health communication [25-27]. For instance, modality interactivity has been found to positively affect attitudes toward health websites [27,28] and the persuasive effect health messages [26]. Furthermore, interactivity has been positively associated with behavioral intentions such as intention to recommend a fitness center [25], as well as intention of actual physical exercise [29].

This study was designed to examine how manipulations in the design and presentation of sun protection health messages impact college students’ sun protection intentions (primary outcome) as well as their attitudes, trust of messages, and information processing (secondary outcomes). Specifically, we examined (1) how 3 experimental message manipulations (ie, human presence in health messages, screen size, and interactivity) influenced the primary outcome and secondary outcomes; and (2) potential interactions in the impact of the experimental messages on both primary and secondary outcomes.

## Methods

### Recruitment and Participants

Participants from a large US university in the American Pacific Northwest were recruited to sign up through a research participation system called Sona system and received course credit as incentive. Study approval was obtained from the university’s institutional review board before data collection.

### Procedures

Participants who signed up for the study were asked to come to a communication laboratory. As the independent variables were between-subjects factors, participants were randomly assigned to 1 of the 8 experimental conditions. Participants in the big screen size conditions were provided with an Apple iPad Air (9.7-inch screen), whereas participants in the small screen size conditions were provided with an Apple iPhone 6 or 6s (4.7-inch screen). After being provided with a mobile device, each participant was instructed to explore a health website and read all the information displayed on the website. When participants finished exploring the health websites, they were instructed to complete a questionnaire in Qualtrics using identical laboratory computers.

### Experimental Treatment Conditions

**Figure 1:**
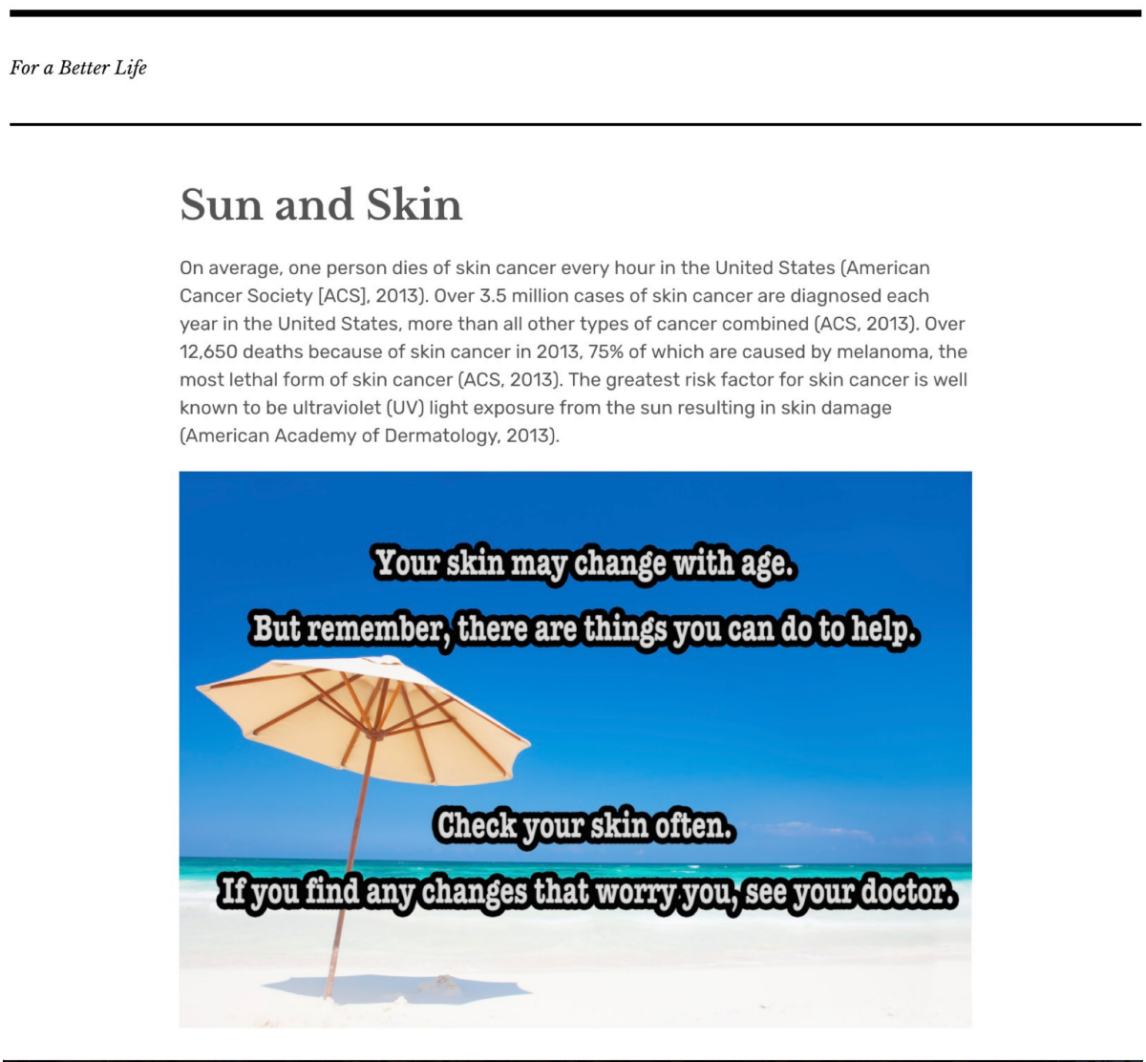
Screenshot of the website homepage.

**Figure 2:**
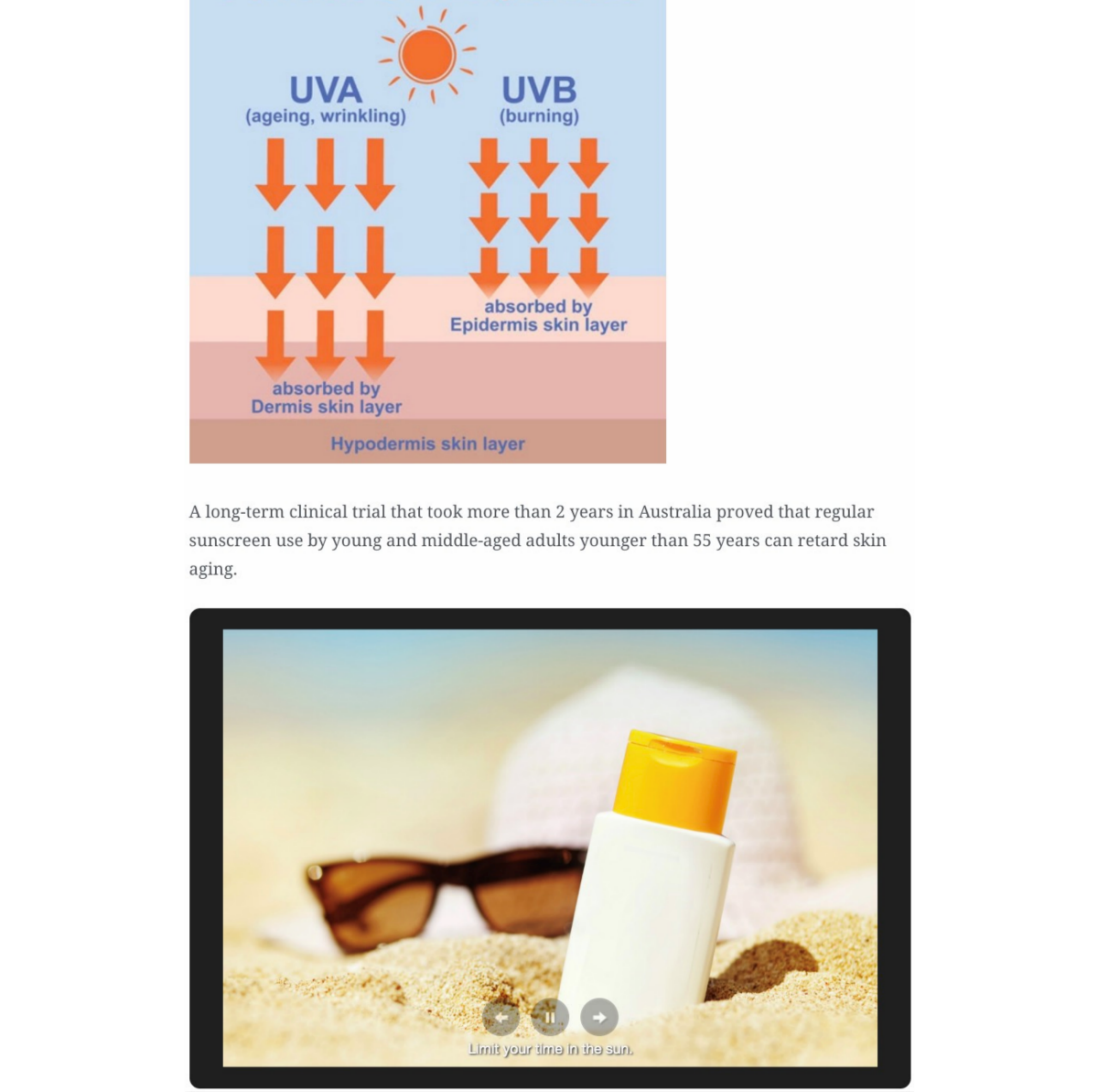
Screenshot of the webpage of high interactivity and human absence condition.

**Figure 3:**
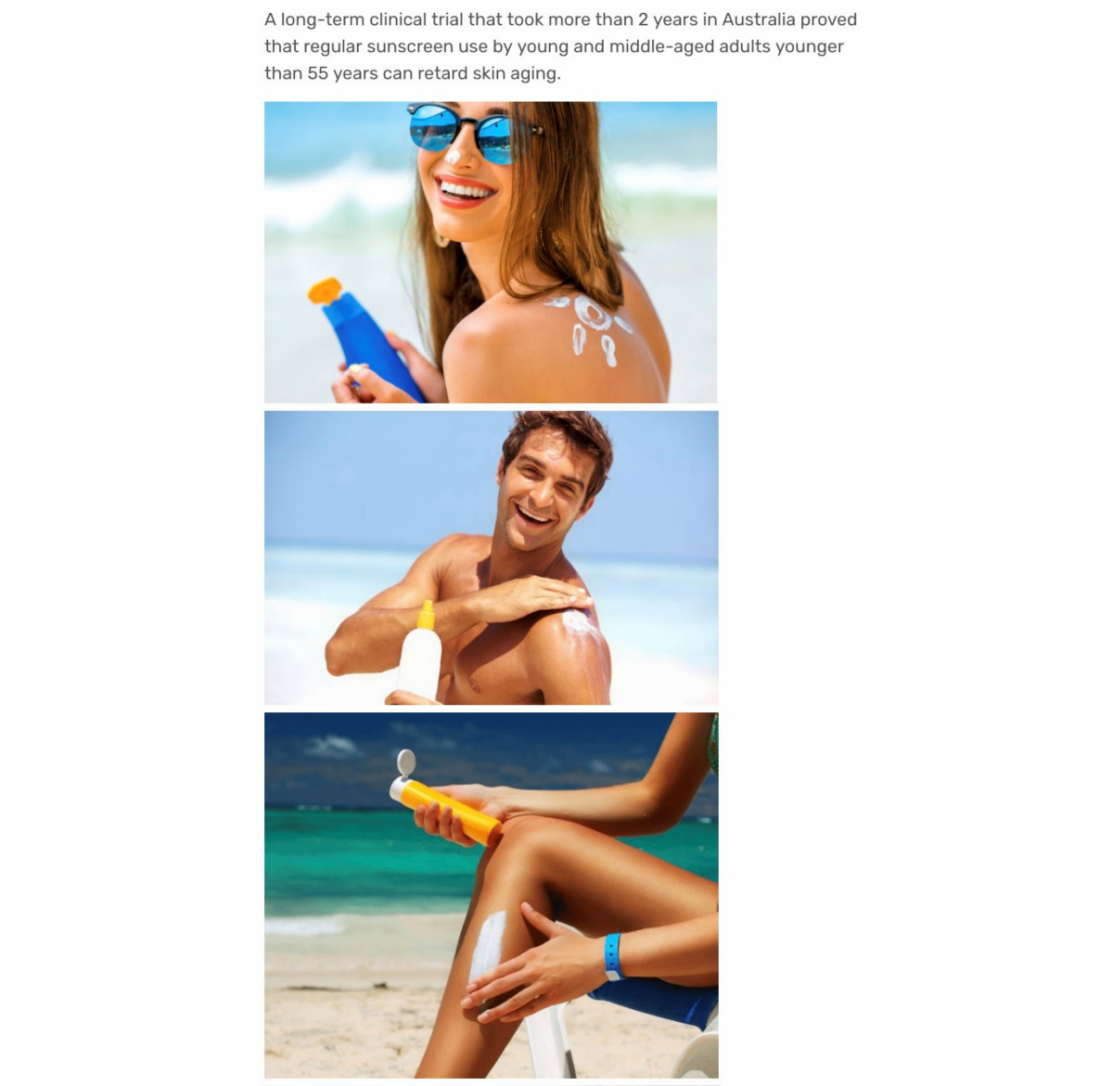
Screenshot of the webpage of low interactivity and human presence condition.

A total of 4 websites were created for the experiment to test the 2 (*human presence*: people vs no people)×2 (*screen size*: big screen vs small screen)×2 (*interactivity*: high vs low) between-subjects design. Although all websites had the same title (*Sun and Skin*), webpage layout (Figure 1), and health information about skin cancer, sunburn, and aging, they differed in terms of interactive features and images with or without human presence. The high interactivity condition included interactive function such as *clicking* and *zooming* that allowed participants to interactively access the website content, whereas the low interactivity condition simply loaded content with minimum participant control. The human presence conditions contained images of humans in relation to sun protection objects, whereas the human absence condition strictly contained the sun protection objects (eg, sunscreen and hat). Screenshots of website pages are shown in Figures 2 and 3 as examples. Screen size was manipulated through mobile devices with different screen sizes.

### Measures

#### Manipulation Checks

Two self-report questions were used as manipulation checks to examine the effectiveness of the manipulations of the experimental conditions. The manipulation of *perceived interactivity* was assessed using 3 items adapted from the study by Kalyanaraman and Sundar [30]. The manipulation of *human presence* was assessed by a 3-point question (1=*disagree*, 2=*neutral*, and 3=*agree*) asking the extent to which the participants agreed that they saw a human figure on the website.

#### Primary Outcome

*Behavioral intentions* were measured with five 5-point Likert-type items reflecting participants’ behavioral intention to use sun protection mentioned in the health message [31], such as “In the future, how often do you intend to use sunscreen with Sun-Protection Factor (SPF) 15 or higher on your face when you were in the sun” (alpha=.80, mean 3.32, SD 0.83).

#### Secondary Outcomes

*Attitudes toward skin cancer message* were measured using five 7-point statements from a reliable scale in previous studies [26,32] such as asking the respondents to “indicate whether you feel that the messages on the website you just viewed was believable or not *”* with response options anchored with *not believable* and *believable*. The remaining items included *not informative* or *informative*, *not insightful* or *insightful*, *not interesting* or *interesting*, and *not clear* or *clear*. Items were averaged to create an attitude index (alpha=.75, mean 5.94, SD 0.77).

*Cognitive trust* was measured using 4 items from previous work on trust and credibility of online information [33,34], asking, “to what extent do you agree or disagree with the following statements: The health information I just read was accurate/accurate/reliable/credible/believable” on a 10-point Likert scale ranging from 1 (*not at all*) to 10 (*extremely*; alpha=.89, mean 7.81, SD 1.65). Similarly, *affective trust* was measured using items from Koh and Sundar’s [34] dimensions of trust that are designed to capture emotion-driven trust of information. Moreover, four 10-point Likert-type items ranging from 1 (*not at all*) to 10 (*extremely*) were used to measure whether the respondents felt that the health information they just read was *likable*, *interested in my well-being*, *empathetic*, and *warm* (alpha=.81, mean 6.79, SD 1.70).

*Heuristic processing* was measured using four 7-point Likert-type items adapted from the study by Griffin et al [35], such as, *When I encounter information about this topic, I focus on only a few key points* (alpha=.71, mean 2.59, SD 0.90). *Systematic processing* was measured using five 7-point Likert-type items adopted from the same questionnaire, such as, “After I encounter information about this topic, I am likely to stop and think about it” (alpha=.78, mean 5.01, SD 1.16).

#### Covariates

Participants who had paid attention on media to skin cancer information, sun protection information, or both types of information are more likely to be familiar with the related information. Thus, *media attention* was used as a covariate in this study. Participants’ *media attention* was adapted from the study by Brossard and Nisbet [36]. Participants were asked to indicate their level of agreement on 4 items with a 7-point scale ranging from 1 (*strongly disagree*) to 7 (*strongly agree*), including items such as “I have paid attention to information related to skin cancer and/or sun protection in the past” (alpha=.91).

### Statistical Analysis

A power analysis was conducted to ensure that the current sample size was sufficient for testing the hypotheses, with a power of 81% to detect a medium effect size for *F* test. A series of analysis of covariance (ANCOVA) tests were used to test the main effects and interaction effects of human presence, interactivity, and screen size on the primary and secondary outcomes. Media attention to skin cancer and/or sun protection information, race or ethnicity (white vs nonwhite), and sex were controlled as covariates in all ANCOVA analyses.

## Results

### Sample

The initial sample consisted of 147 undergraduate students, but data cleaning yielded an analytical sample size of 134 participants (cases with missing data were excluded). The mean age of the participants was 19.94 years (SD 2.22). More than 60.0% of the sample was female (83/134, 61.9%). Participants identified as white (76/134, 56.7%), black (15/134, 11.2%), Hispanic (19/134, 14.2%), Asian (15/134, 11.2%), and other (9/134, 6.7%).

### Manipulation Checks

Independent sample *t* test was used to check the manipulation of interactivity. According to the results, participants in high interactivity condition (mean 3.25, SD 0.98) scored higher on perceived interactivity than participants in low modality condition (mean 2.87, SD 1.08, *t*_132_=2.31; *P*=.04). Similarly, participants in human presence condition (mean 2.71, SD 0.68) scored higher on seeing people in the pictures than participants in human absence condition (mean 1.08, SD 0.41, *t*_132_=17.03; *P*<.001). Results of all ANCOVA tests are presented in Table 1.

**Table 1:** Results of analysis of covariance tests (6 rows represent six 3-way analyses of covariance tests).

Outcome	Covariates^a^			Experimental manipulations^a^			Interactions^a^			
	Sex	Race or ethnicity	Media attention	Human presence	Screen size	Interactivity	Human presence × screen size	Human presence × interactivity	Screen size × interactivity	3-way interaction
Behavioral intentions	.02^b^	.09	.67	<.001^b^	<.001^b^	<.001^b^	.15	.008^b^	<.001^b^	.02^b^
Attitudes	.06	.31	.26	.02^b^	.47	.12	.13	.23	.02^b^	.48
Affective trust	.98	.76	.10	.006^b^	<.001^b^	.12	.90	.52	.61	.95
Cognitive trust	.74	.09	.71	<.001^b^	.007^b^	.64	.35	.16	.48	.25
Systematic processing	.06	.81	.51	.34	.04^b^	.70	.34	.59	.85	.13
Heuristic processing	.16	.55	.84	.07^b^	.03^b^	.23	.73	.49	.38	.20

^a^Values are *P* values of the tests.

^b^Values less than .05 indicate statistical significance.

### Behavioral Intentions

#### Main Effects

Human presence had a main effect on behavioral intentions (*F*_1,123_=14.90, *P*<.001, η_p_=0.11); participants in the human presence condition (mean 3.50, SD 0.62) had greater intention to use sun protection than did participants in the human absence condition (mean 3.12, SD 0.98). The main effect of screen size was also significant on behavioral intentions (*F*_1,123_=25.34, *P*<.001, η_p_=0.17). Participants in the large screen size condition (mean 3.56, SD 0.68) had greater intention to use sun protection than did participants in the small screen size condition (mean 3.02, SD 0.91). Interactivity also demonstrated a main effect on behavioral intentions (*F*_1,123_=14.37, *P*<.001, η_p_=0.11). Participants in the high interactivity condition (mean 3.46, SD 0.68) had greater intention to use sun protection than did participants in the low interactivity condition (mean 3.16, SD 0.96). Female participants were more likely to use sun protection than male participants (*P*=.02).

#### Interaction Effects

**Figure 4:**
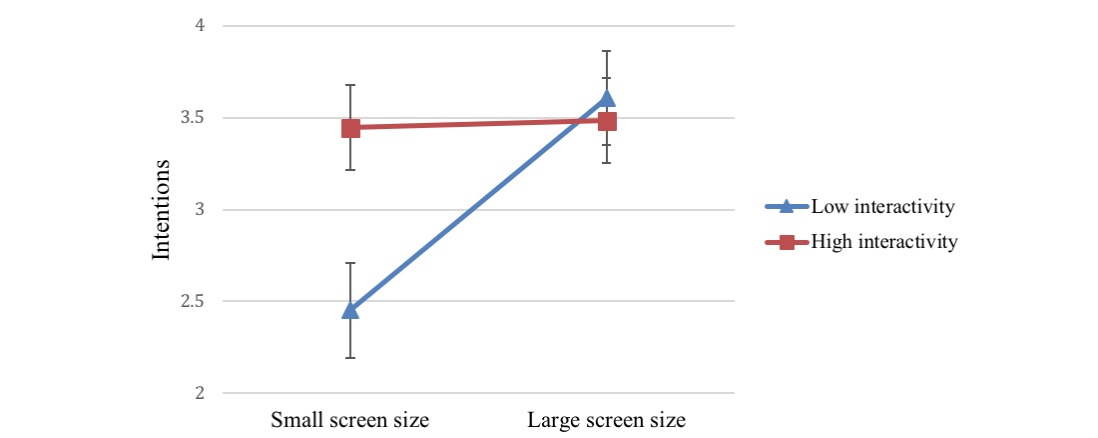
Two-way interaction of screen size and interactivity on intentions.

There was a significant 2-way interaction effect of screen size and interactivity on behavioral intentions (*F*_1,123_=23.75, *P*<.001, η_p_=0.16). Large screen size increased the effects of interactivity (high: mean 3.48, SE 0.11; low: mean=3.61 *,* SE 0.11) on behavioral intentions (Figure 4).

**Figure 5:**
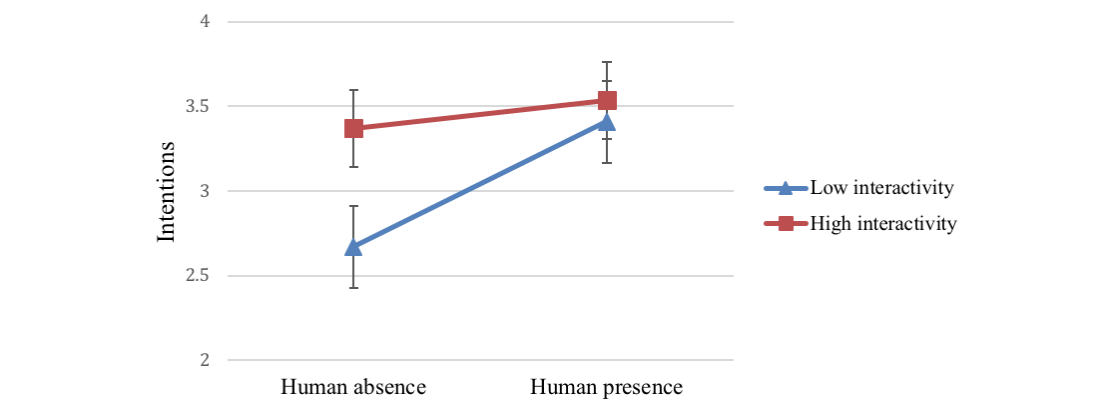
Two-way interaction of human presence and interactivity on intentions.

Furthermore, there was also a significant 2-way interaction effect of human presence and interactivity on behavioral intentions (*F*_1,123_=7.17, *P*=.008, η_p_=0.06), indicating human presence helped reinforcing the effects of interactivity (high: mean=3.54 *,* SE 0.11; low: mean 3.41 *,* SE 0.12) on behavioral intentions. This indicates that although human absence and low interactivity individually led to diminished intentions to act on the message, coupling these with high interactivity and human presence mitigated these effects and positively influenced and intentions to act (Figure 5).

**Figure 6:**
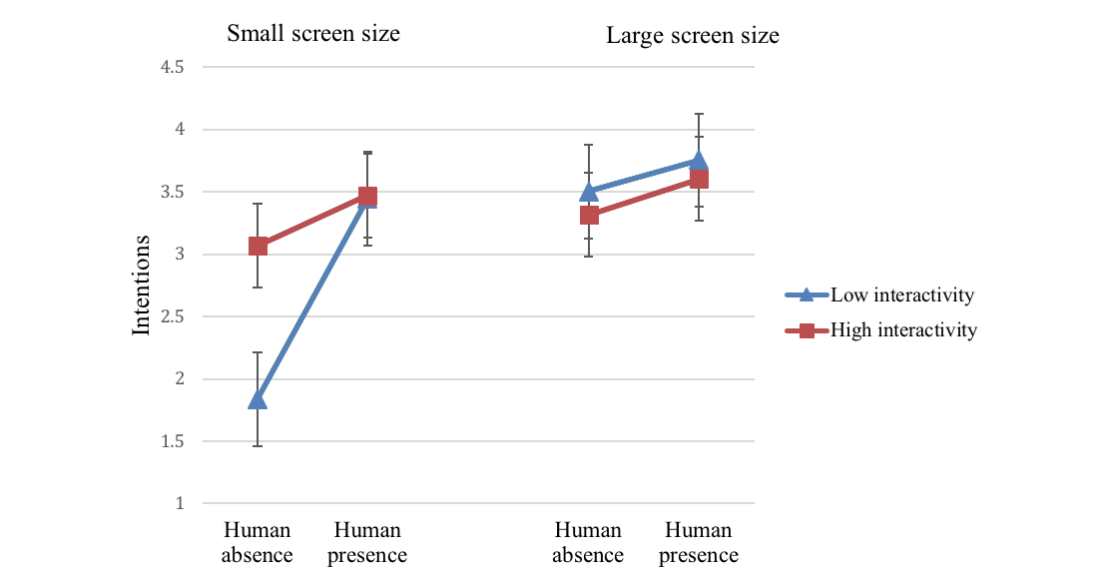
Three-way interaction of screen size, interactivity, and human presence on intentions.

Finally, a significant 3-way interaction effect of screen size, human presence, and interactivity on behavioral intentions was found (*F*_1,123_=5.43, *P*=.02, η_p_=0.04; Figure 6). Human presence increased behavioral intentions in the small screen size conditions for both high and low interactivity such that highest behavioral intention scores were reported among those who viewed websites with human presence on small screen size devices with high interactivity (mean 3.47, SE 0.16) or low interactivity (mean 3.44, SE 0.17). In the large screen size condition, behavioral intention scores were the highest in the human presence condition with high (mean 3.61, SE 0.15) or low interactivity (mean 3.76, SE 0.16). In conclusion, human presence had a consistently positive impact on intentions across different levels of interactivity and screen size. Specifically, there was a marked difference in intention to use sun protection, depending on the low and high interactivity in the absence of a human presence on a small screen.

### Attitudes

#### Main Effect

Only human presence had a main effect on attitudes toward health information (*F*_1,123_=6.10, *P*=.02, η_p_=0.05); participants in the human presence condition (mean 6.10, SD 0.75) had more favorable attitudes toward health information than participants in the human absence condition (mean 5.76, SD 0.76).

#### Interaction Effects

**Figure 7:**
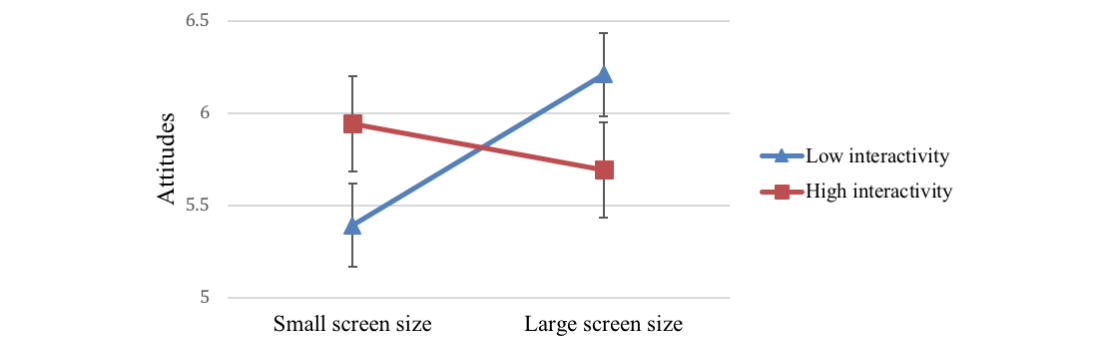
Two-way interaction of screen size and interactivity on attitudes.

A significant 2-way interaction effect of screen size and interactivity on attitudes toward health information was observed (*F*_1,123_=5.72, *P*=.02, η_p_=0.04). Large screen size had stronger effects on attitudes in the low interactivity condition (mean 6.23, SE 0.12), whereas small screen size had stronger effects on attitudes in high interactivity condition (mean 5.93, SE 0.13; Figure 7). These results indicated that although small screens and low interactivity individually led to diminished attitudes toward the health message and intentions to act on the message, coupling these with high interactivity and large screen sizes mitigated these effects and positively influenced attitudes and intentions.

### Affective and Cognitive Trust

There was a significant effect of human presence on affective trust (*F*_1,123_=7.93, *P*=.006, η_p_=0.06) and cognitive trust (*F*_1,123_=13.22, *P*<.001, η_p_=0.10). In addition, screen size was observed to have a main effect on multidimensional trust. Large screen size led to more affective trust (*F*_1,123_=13.57, *P*<.001, η_p_=0.10); participants in the large screen size condition (mean 7.22, SE 1.74) reported higher affective trust than small screen size condition (mean 6.23, SE 1.50). Small screen size led to more cognitive trust (*F*_1,123_=7.64, *P*=.007, η_p_=0.06); participants in the small screen size condition (mean 8.28, SE 1.53) reported higher affective trust than large screen size condition (mean 7.52, SE 1.60). No interaction effects were found for affective or cognitive trust.

### Information Processing

Screen size had a significant effect on heuristic-systematic processing. Large screen size led to more heuristic processing (*F*_1,123_=4.74, *P*=.03, η_p_=0.04), and small screen size led to more systematic processing (*F*_1,123_=4.22, *P*=.04, η_p_=0.03). Participants who viewed information on large screen devices reported higher heuristic processing (mean 2.74, SE 0.99) than those in small screen size condition (mean 2.39, SE 0.76). Participants in the small screen size (mean 5.28, SE 0.88) condition had higher systematic processing than participants in the large screen size condition (mean 4.82, SE 1.29). This indicates that screen size did differ on influencing heuristic-systematic information processing. No interaction effects were found for heuristic or systematic processing.

## Discussion

### Principal Findings

This study was an experimental study that aimed to examine the impact of a mobile-based educational health program on promoting sun protection behavioral intentions among college students. The individual and combinatory effects of technological factors, such as screen size and interactivity, and social factors, such as human presence human on the primary outcome including intention to use sun protection, and secondary outcomes including attitudes toward the message, trust of the message, and information processing were examined. The preliminary results regarding increased intention to use sun protection supported the promising influences of the educational program, and the results also demonstrated implications for the design of a future sun protection intervention delivered through mobile technologies among college students.

Generally, it was found that the presence of human characters in the educational message was very effective in garnering favorable attitudes, trust, and intentions to act on the sun protection advice in the message. More specifically, the presence of human characters influenced one’s affective trust, one’s attitude toward the educational message, and one’s intention to use sun protection as promoted in the message. This finding regarding the effects of human presence on attitudes, trust, and intentions is consistent with previous research about the role of presence in advertising, online shopping, and social commerce [37-39], which suggested the persuasive role of human presence in health behavior science. It was also found that delivering educational health messages on a large iPad screen led to greater intentions, greater affective trust, and heuristic processing, whereas messages on a smaller iPhone screen led to cognitive trust and systematic processing. These results are consistent with previous literature regarding the effects of presentation mode on trust [20]. Although higher interactivity did not lead to more favorable attitudes toward the message, it did lead to greater intentions to act on the educational message, confirming the impact of digital interactivity on health-related behavioral outcomes [26].

According to the results of the 2-way interaction effects, attitudes toward the message and intention to use sun protection were positively affected by low interactivity and large screen size. High interactivity and human presence together exerted the highest scores for behavioral intention to use sun protection. The 3-way interaction suggested that behavioral intention was consistently and positively impacted by human presence across the different levels of interactivity and screen size. Interactivity level did not make a huge difference when 3 factors were present together. However, there was a marked difference in intention to use sun protection between the interaction of low interactivity and absence of a human character on a small screen and the interactions of other combinations.

In sum, this study suggests that mHealth programs may increase health outcomes by not only manipulating the screen size and implementing interactivity modalities but also by placing an emphasis on social factors that represent human presence. This human presence serves to both engage the user in a communicative perspective and act as a physical model for actionable behavioral outcomes, such as applying sunscreen. Furthermore, the results demonstrate the significance of screen size in the effectiveness of mobile-based educational program. It is also worth noting that the larger screen size would increase the size of the human characters represented in the educational materials, which may augment the impact of human presence. As the results suggest, a small screen requires the educational materials to be combined with other factors to be more effective. For instance, although small screens are effective for learning when implementing both high interactivity and human presence, these effects are mitigated with low interactivity and in the absence of human characters.

These findings of this study suggest that effective digital education promoting health-related outcomes should include human characters, a large screen size, and some level of modality interactivity. Although mobile device use is ubiquitous among the study sample of college students, it is unclear how different mobile device types may influence college students’ cognitive and behavioral intentions and attitudes toward health-related educational content [8,40-42]. A clear implication of this study is that health practitioners/educators designing health educational messages using mobile technologies must account for the effectiveness and form of educational messages across different devices and the suitability of these different messages for students with different access to different mobile technologies.

### Limitations and Future Directions

This study also had a number of limitations. First, although the study was adequately powered to test for message effects on the outcomes of interest, the small sample size leads to concerns about the generalizability of results from this single study. In addition, the observation of statistically significant differences in outcomes of interest in an experimental study does not imply that changing intentions or message processing outcome would necessarily produce clinically significant changes in sun protection behavior. A future formal health intervention with a large sample is needed to examine the efficacy of mHealth programs designed based on the findings of this study and their ability to produce meaningful behavioral changes among college students. Moreover, the human presence condition was rather simplistic, essentially referring to the physical presence or absence of a human character. Future work should explore this variable with greater degree of gradation, such as placing individuals in different situational and contextual environments, comparing the effects across different actions performed by human characters, and comparing the effects of multiple human characters included in a single educational setting. Furthermore, the human characters represented in the stimuli materials were depicted in still images. The effectiveness of human characters may be more refined by exploring the impact of physical movement and voice. In addition, the effects for manipulating the presentation of human characters across sex, race, and ethnicity should also be explored in future work, particularly for different groups of subjects.

Furthermore, this study did not control for participants’ familiarity with the devices used in the experiment. For instance, users of iPads may be more or less acceptant of messages delivered on iPads. Alternatively, users may have psychological attachment to certain devices, which may have a stronger influence than the size of the screen. Critically, certain groups of individuals may not have access to devices used in the study, which could impact their responsiveness to the content delivered on the devices. Future work would benefit from exploring these device-specific complexities, as this would prevent a bias toward users who have access to and familiarity with such a high-cost device.

Finally, the health information was delivered on a mobile version website on the mobile devices. Future studies can investigate whether participants prefer receiving health information on mobile apps or a website on mobile devices.

### Conclusions

This study aimed to investigate how mobile digital technologies and a social factor of a mobile-based educational program may influence health-related outcomes. The observed results suggest feature-specific recommendations for educational program design. Specifically, results indicate that intention to use sun protection remains relatively constant across levels of interactivity and human presence on a large screen, whereas the negative impact of low interactivity is more pronounced on a smaller screen. In sum, this study suggests social factors should be integrated with digital technologies to maximize the effects when designing and delivering health-related educational messages in mobile-based programs that are aiming at generating actionable intent for health behavior.

## Abbreviations

ANCOVA: analysis of covariance
mHealth: mobile health
UV: ultraviolet
